# RuvBL2 Is Involved in Histone Deacetylase Inhibitor PCI-24781-Induced Cell Death in SK-N-DZ Neuroblastoma Cells

**DOI:** 10.1371/journal.pone.0071663

**Published:** 2013-08-16

**Authors:** Qinglei Zhan, Sauna Tsai, Yonghai Lu, Chunmei Wang, Yiuwa Kwan, Saiming Ngai

**Affiliations:** 1 Centre for Soybean Research of Partner State Key Laboratory of Agrobiotechnology and School of Life Sciences, The Chinese University of Hong Kong, Shatin, New Territories, Hong Kong SAR, China; 2 School of Biomedical Sciences, Faculty of Medicine, The Chinese University of Hong Kong, Shatin, New Territories, Hong Kong SAR, China; University of Miami School of Medicine, United States of America

## Abstract

Neuroblastoma is the second most common solid tumor diagnosed during infancy. The survival rate among children with high-risk neuroblastoma is less than 40%, highlighting the urgent needs for new treatment strategies. PCI-24781 is a novel hydroxamic acid-based histone deacetylase (HDAC) inhibitor that has high efficacy and safety for cancer treatment. However, the underlying mechanisms of PCI-24781 are not clearly elucidated in neuroblastoma cells. In the present study, we demonstrated that PCI-24781 treatment significantly inhibited tumor growth at very low doses in neuroblastoma cells SK-N-DZ, not in normal cell line HS-68. However, PCI-24781 caused the accumulation of acetylated histone H3 both in SK-N-DZ and HS-68 cell line. Treatment of SK-N-DZ with PCI-24781 also induced cell cycle arrest in G2/M phase and activated apoptosis signaling pathways via the up-regulation of DR4, p21, p53 and caspase 3. Further proteomic analysis revealed differential protein expression profiles between non-treated and PCI-24781 treated SK-N-DZ cells. Totally 42 differentially expressed proteins were identified by MALDI-TOF MS system. Western blotting confirmed the expression level of five candidate proteins including prohibitin, hHR23a, RuvBL2, TRAP1 and PDCD6IP. Selective knockdown of RuvBL2 rescued cells from PCI-24781-induced cell death, implying that RuvBL2 might play an important role in anti-tumor activity of PCI-24781 in SK-N-DZ cells. The present results provide a new insight into the potential mechanism of PCI-24781 in SK-N-DZ cell line.

## Introduction

Neuroblastoma is the most common extracranial solid tumor in children and a major cause of neoplastic death in infancy. It accounts for more than 7% of tumors in patients younger than 15 years and causes 15% of deaths in pediatric oncology [1]. The tumor arises from aberrant sympathetic nervous system. It has been reported that common DNA variations are a significant contribution to the development of disease [2]. Therefore, analysis of DNA variations can be used to predict disease progression [3]. Current surgery and radiotherapy in conjunction with chemotherapy has greatly improved survival rates for the patients with low-risk and intermediate-risk neuroblastoma. However, high-risk patients still have an overall survival rate of less than 40% despite intensive therapy [4]. Relapse inevitably occurs in 50%–60% of patients with high-risk neuroblastoma due to acquired drug resistance [2]. Thus, it is urgent to develop new drugs to treat high-risk neuroblastoma.

Histone deacetylase (HDAC) inhibitors have emerged as promising therapeutic agents for cancer treatment due to their low toxicity toward normal cells [5], [6]. Increasing evidence has been shown that epigenetic regulations including DNA methylation and histone modifications could affect changes in chromatin structure, subsequently leading to diverse patterns of gene expression [7]. It has been commonly accepted that aberrant epigenetic regulations contribute to tumorigenesis [8]. A genome-wide study on epigenetic changes in cancer has found that the global loss of acetylation of histone H4 might be a common hallmark in human cancer cells [9]. The hypoacetylation status in cancer cells could be potentially reversed, triggering the development of HDAC inhibitors. Such HDAC inhibitors demonstrated powerful anticancer activity in many types of tumors while displaying limited cytotoxicity in normal cells. Most of them are currently in clinical trials [10]. Vorinostat was the first HDAC inhibitor approved by the Food and Drug Administration (FDA) in 2006 for the treatment of cutaneous T-cell lymphoma [11]. HDAC inhibitors can induce a range of biological responses in tumor cells, such as differentiation, cell cycle arrest, mitotic failure and cell death via apoptosis, autophagy or necrosis [12], [13], [14], [15], [16].

Several studies have shown that HDAC inhibitors such as sodium butyrate (NaB), suberoylanilide hydroxamic acid (SAHA) and trichostatin A (TSA) significantly inhibited neuroblastoma cell growth [17], [18], [19]. Cell cycle arrest in G1/S or G2/M phase was described in some neuroblastoma cell lines after treatment with HDAC inhibitors [20], [21]. The HDAC inhibitor carboxycinnamic acid bis-hydroxamide (CBHA), in combination with retinoic acid synergistically suppressed tumor growth using a human neuroblastoma xenograft in vivo [22]. Multiple mechanisms have been proposed to explain the potent anticancer activity of HDAC inhibitors in neuroblastoma cells. For example, the effect of a HDAC inhibitor VPA on apoptosis was mediated by repression of survivin and Akt pathway [23]. In addition to histones, HDACs also target numerous non-histone proteins such as Ku70, p53 and HSP90 [24]. Upon HDAC inhibitor treatment, the acetylated Ku70 could translocate Bax from cytosol to mitochondria, leading to caspase-dependent apoptosis in N-type neuroblastoma cells [25]. Furthermore, HDAC6 was shown to regulate the interaction between Ku70 and Bax in neuroblastoma cells [26]. A recent study has indicated that vorinostat could enhance neuroblastoma radiotherapy with ^131^I-MIBG via increased expression of the norepinephrine transporter, an uptake protein for ^131^I-MIBG [27].

PCI-24781 is a novel hydroxamic acid-based HDAC inhibitor that shows very promising efficacy and safety in vitro and in vivo for cancer treatment [28]. In this study, the mechanisms of PCI-24781-induced cell death were investigated in neuroblastoma cells. We show here that PCI-24781 exhibits significant anti-tumor activity in SK-N-DZ neuroblastoma cells. PCI-24781 caused cell cycle arrest in G2/M phase and apoptosis in SK-N-DZ cells not in HS-68 normal cells, although both acetylated H3 was accumulated in response to PCI-24781. Our further proteomic analysis identified a total of 42 differentially expressed proteins that involved in multiple biological processes including signal transduction, transcriptional regulation, metabolism, cell cycle and proliferation. Moreover, the effect on cell death induced by PCI-24781 is possibly mediated via RuvBL2, an AAA+ ATPase, since knockdown of RuvBL2 can partially rescue cells from apoptosis. We thus provide new information about the mechanism of action of PCI-24781.

## Materials and Methods

### Cell Culture and Reagents

A human normal foreskin fibroblast cell line HS-68 and three human malignant neuroblastoma cell lines (SK-N-DZ, SH-SY-5Y and SK-N-SH) were purchased from American Type Culture Collection (ATCC, Rockville, MD, USA). Cells were cultured in DMEM supplemented with 10% FBS (Hyclone, Logan, UT), 100 U/ml penicillin, and 0.1 mg/ml streptomycin (GIBCO, Grand Island, NY) and maintained at 37°C in a humidified 5% CO_2_ incubator. The HDAC inhibitor PCI-24781 was obtained from Selleckchem (Houston, TX) and CI-994 was purchased from Sigma-Aldrich (St. Louis, MO). All drugs were dissolved in DMSO to create stock solutions. All serial dilutions were carried out using cell culture medium.

### Measurement of Cell Viability

Cell viability was evaluated by MTT (3-(4, 5-dimethylthiazol-2-yl)-2, 5-diphenyltetrazolium) assay. Cells (1–2.5×10^4^/well) were plated in 96 well plates for 24 h and then incubated with different concentrations of PCI-24781 and CI-994 for additional 24 h, in a final volume of 200 µl. After treatment, each well was incubated with 20 µl of MTT (5 mg/ml in PBS) for 4 h at 37°C. The resulting purple formazan was dissolved in 200 µl DMSO, and the absorbance was measured at 570 nm on a BioTek microplate reader (Winooski, Vermont). Cell viability was expressed as percentages. The absorbance measured from non-treated cells was considered as 100%. Data are shown as the mean ± SD of triplicate assays.

### Apoptosis and Cell Cycle Analysis

Cells were collected after the indicated treatment with PCI-24781, and washed twice with ice-cold PBS. Cells were then fixed with 70% ice-cold ethanol for at least 1 h at 4°C. For staining with propidium iodide (BD Biosciences, San Diego, CA), cells were centrifuged and the pellet was resuspended in 500 µl of propidium iodide containing RNase. The stained cells were filtered with 41 µm membrane (Millipore, Billerica, MA) and then analyzed by FACS flow cytometer (Beckman Coulter, Fullerton, CA). Three independent experiments were performed. At least 10,000 cells from each sample were analyzed, and the percentages of cells in sub-G_1_, G_1_, S and G_2_/M phases were calculated with CXP software.

### Apoptosis Signaling Pathways

Total 200 µg of cell lysates from untreated and treated cells were incubated with human apoptosis antibody array membranes (RayBio-tech, Norcross, GA) according to the manufacturer’s instructions. Band development was detected by using chemiluminescence method.

### Immunoblotting Analysis

After treatments as indicated, total cell lysates were prepared, and 40 µg of protein was separated by 12% SDS-PAGE. For histone preparation, cells were washed with ice-cold PBS for twice, and then isolated using salt-urea method as described [29]. Three microgram of histone was subjected to 15% SDS-PAGE. The proteins were then transferred to PVDF membranes and incubated with specific antibodies. Bound antibodies were detected by chemiluminescence (Cell Signaling, Danvers, MA) according to manufacturer’s instructions. The following primary antibodies and dilutions were used for Western blotting analyses: H3 (1∶2000), p21 (1∶500), DR4 (1∶500), GAPDH (1∶2000), hHR23a (1∶6000), RuvBL2 (1∶2000), Ku70 (1∶200), Rad51 (1∶2000), PDCD6IP (1∶1000) and TRAP1 (1∶10000) from Abcam (Cambridge, MA); p53 (1∶1000), caspase 3 (1∶1000), cleaved caspase 3 (1∶1000) from Cell Signaling; prohibitin (1∶2000) from Santa Cruz Biotechnology (Santa Cruz, CA); acetylated H3 (1∶6000) from Millipore.

### 2-DE and Image Analysis

Cells were lysed in 1 ml lysis buffer (7 M urea, 2 M thiourea, 4% CHAPS) containing protease inhibitor cocktail and PMSF (Sigma). Samples were then kept on ice for 30 min with occasional vortex mixing. After centrifugation at 12,000 g for 1 h at 4°C, supernatants were precipitated with cold 80% acetone containing 0.05% acetic acid at −20°C overnight and then dissolved with rehydration buffer (8 M urea, 2 M thiourea, 4% CHAPS, 100 mM DTT). Protein concentrations were quantified by 2-D Quant kit (GE Healthcare, Piscataway, NJ) according to the manufacturer’s instructions. Total proteins of 500 µg were applied to an immobilized pH gradient (IPG) strip (13 cm, pH 3–10 NL, GE Healthcare). The strips were then subjected to isoelectric focusing (IEF) and the total time×voltage was 40000 Vh for each strip. The second dimensional separation was performed using 12% SDS-PAGE after equilibration. The gels were stained by Coomassie Brilliant Blue (CBB) R-250 (Sigma) and then scanned. At least triplicate independent runs were carried out to ensure accuracy. For spots detection and quantification, the images were analyzed by the ImageMaster™ software version 5.0. Only spots with ratios above 1.5 and under 0.5 when compared to the control group were selected for MS analysis.

### In-gel Digestion

Protein spots of interest were excised from the gels using needle, and destained with 25 mM ammonium bicarbonate (NH_4_HCO_3_)/50% methanol. After washing in water once, spots were dehydrated in 100 µl 100% ACN thrice and then dried in vacuum pumps (Labconco, Kansas, MO). The gels were then digested with 20 ng/µl trypsin in 25 mM NH_4_HCO_3_ for 16 h at 30°C. Tryptic samples were extracted with 80% ACN/2.5% trifluoroacetic acid (TFA) by sonication for 10 min, and centrifuged at 12,000 g for 1 min. The supernatants were then subjected to MS analysis.

### MALDI-TOF MS Analysis and Protein Identification

For MS analysis, 0.5 µl of the tryptic peptides were spotted onto a 96-well target MALDI plate in triplicate and followed by 0.5 µl of α-cyano-4-hydroxycinnamic acid (Sigma). Mass spectra were acquired using matrix-assisted laser desorption/ionization time-of-flight mass spectrometry (MALDI-TOF MS) system from a 4700 Proteomics Analyzer (Applied Biosystems, USA). The top eight most abundant ions for each MS scan were selected for MS/MS analysis. The MS/MS data were then analyzed by using GPS Explore™ software (Applied Biosystems) and MASCOT search engine. Database searches were performed using the following parameters: database, NCBInr; taxonomy, *Homo sapien*; enzyme, trypsin; precursor tolerance, 50 ppm; MS/MS ion tolerance, 0.1 Dalton; peptide charge, 1+; and allowance of one missed cleavage. Oxidation of methionine was selected as a variable modification. Proteins with scores of C.I. % ≥ 95% were considered to be successfully identified.

### RNA Interference

Cells were seeded into six-well plates at 2×10^5^ cells per well and cultured in medium without antibiotics. After 24 h, cells were transfected with siRNAs at a final concentration of 5 nM using Lipofectamine RNAiMAX (Ambion, Carlsbad, CA). Commercially available Silencer Select Pre-designed siRNAs for RuvBL2, hHR23a and a negative control (Ambion) were used. The sequences of siRNAs for RuvBL2 and hHR23a were 5′-GAAACGCAAGGGUACAGAAtt-3′ and 5′-GAACUUUGAUGACGAGUGAtt-3′, respectively. Cells were then exposed to 0.5 µM PCI-24781 for 24, 36 and 48 h after transfection for 48 h.

### Statistics

Dose-response curves and histogram for flow cytometer analysis were generated using GraphPad Prism 6.0 (GraphPad software, San Diego, CA). Band intensities were determined by ImageJ v1.4 software (Bethesda, Maryland), and fold change was calculated by comparing with controls. The data presented are the mean values from three independent experiments ± SD. *P* values used for significance are indicated in the figure legends.

## Results

### HDAC Inhibitors Enhanced Human Neuroblastoma Cell Death

**Figure 1:**
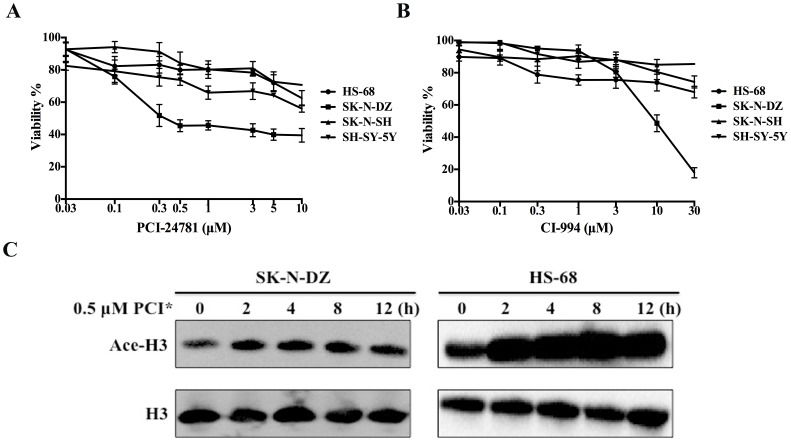
HDAC inhibitors enhanced human neuroblastoma cell death and PCI-24781 caused the accumulation of acetylated histone H3. Three human neuroblastoma cell lines SK-N-DZ, SK-N-SH and SH-SY-5Y and a normal cell line HS-68 were treated with increasing doses of PCI-24781 (A) and CI-994 (B) as indicated for 24 h, respectively. Cell viability was measured by MTT assay. Error bars represent the means ± S.D. of three independent experiments. (C) The changes in histone acetylation level were detected by Western blotting using antibodies specific for acetylated H3 (Ace-H3) and total H3 (H3). PCI* represents PCI-24781.

Previous studies have indicated that PCI-24781 and CI-994 had antiproliferative effects on multiple solid tumors [28], [30]. However, the studies in neuroblastoma cells are limited. The effects of HDAC inhibitors on cell viability were measured by MTT assay in three human neuroblastoma cell lines SK-N-DZ, SK-N-SH and SH-SY-5Y and a normal cell line HS-68 by exposing them to 0.03–10 µM of PCI-24781 and 0.03–30 µM of CI-994 for 24 h, respectively. As shown in Figure 1A, PCI-24781 induced significant cell death in SK-N-DZ and SH-SY-5Y cell lines, and had less effects on SK-N-SH and HS-68 cell lines. Especially for SK-N-DZ cell line, the percentage of viability was less than 50% at the 0.5 µM dose of PCI-24781 after treatment for 24 h. However, there was a significant reduction in SK-N-SH cell viability after exposure to the same dosage of PCI-24781 for 48 h; less effect was observed in HS-68 even treatment for 48 h (Figure S1). CI-994 also induced dose-dependent inhibition in SK-N-DZ cell growth, and less than 50% of viability was observed under the treatment by 10 µM CI-994. On the other hand, two other NB cell lines (SK-N-SH and SH-SY-5Y) and HS-68 cell line were less sensitive to CI-994 treatment (Figure 1B). Based on these results, the NB cell line SK-N-DZ was selected to do further studies due to the most sensitivity to PCI-24781. Moreover, we chose 0.5 µM PCI-24781 for the following experiments, as the cell viability reduced to 45% in SK-N-DZ cells and only to 80% in HS-68 cells at such concentration.

### PCI-24781 Increased the Level of Acetylated Histone H3 Both in SK-N-DZ and HS-68 Cells

To assess the effect of PCI-24781 on acetylation of histone H3 in SK-N-DZ and HS-68 cell lines, cells were treated with 0.5 µM PCI-24781 from 2 to 12 h. Figure 1C showed that PCI-24781 caused the accumulation of acetylated histone H3 between 2 h and 12 h of treatment in SK-N-DZ cell line. Interestingly, the level of acetylated histone H3 was also increased in HS-68 cell line by PCI-24781. The high acetylation level even maintained till 12 h after PCI-24781 treatment.

### PCI-24781 Induced G2/M Cell Cycle Arrest and Enhanced Apoptosis in SK-N-DZ Cell Line

**Figure 2:**
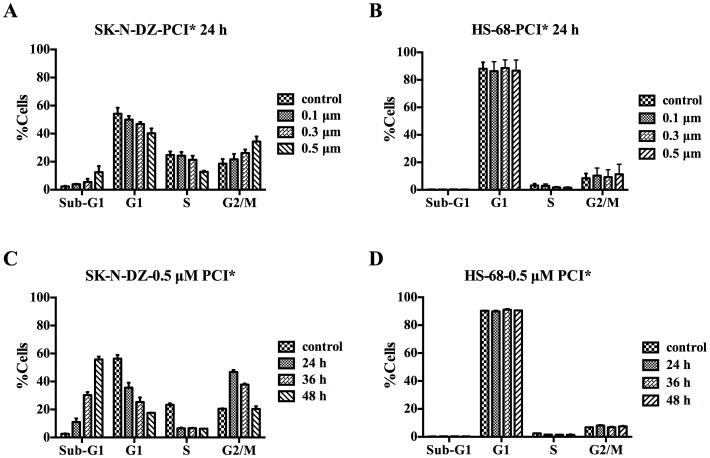
PCI-24781 induced G2/M cell cycle arrest and apoptosis in SK-N-DZ not HS-68 cells. SK-N-DZ and HS-68 cells were treated with the increasing doses of PCI-24781 for 24 h (A and B), and different time points of 0.5 µM PCI-24781 as indicated (C and D), respectively. Cell cycle analysis was performed by propidium iodide staining method. Values are the mean of three independently experiments. Control: no treatment; PCI*: PCI-24781.

As HDAC inhibitors are known to affect cell cycle and/or apoptosis, we then analyzed the cell cycle progression and induction of apoptosis in SK-N-DZ treated with PCI-24781 by flow cytometry. After 24 h treatment, PCI-24781 increased the percentage of SK-N-DZ cells in G2/M phase, indicating a G2/M cell cycle arrest (Figure 2A). Significant time-dependent S phase depletion and G2/M cell cycle arrest was also observed in 0.5 µM PCI-24781 treated cells (Figure 2C). In addition, propidium iodide analysis showed increased accumulation in the sub-G1 cell fraction after PCI-24781 treatment, suggesting an induction of apoptosis in SK-N-DZ cells. When compared with control (no treatment), the percentages of sub-G1 cells were 11%, 31% and 58% after 0.5 µM of PCI-24781 exposure for 24 h, 36 h and 48 h, respectively (Figure 2C). To evaluate whether these cell cycle changes were neuroblastoma cell specific, the effects on HS-68 cells treated with PCI-24781 were also examined. However, no significant dose- or time-dependent cell cycle arrest was observed, and even no evident sub-G1 peak occurred after PCI-24781 treatment as indicated (Figure 2B and D).

**Figure 3:**
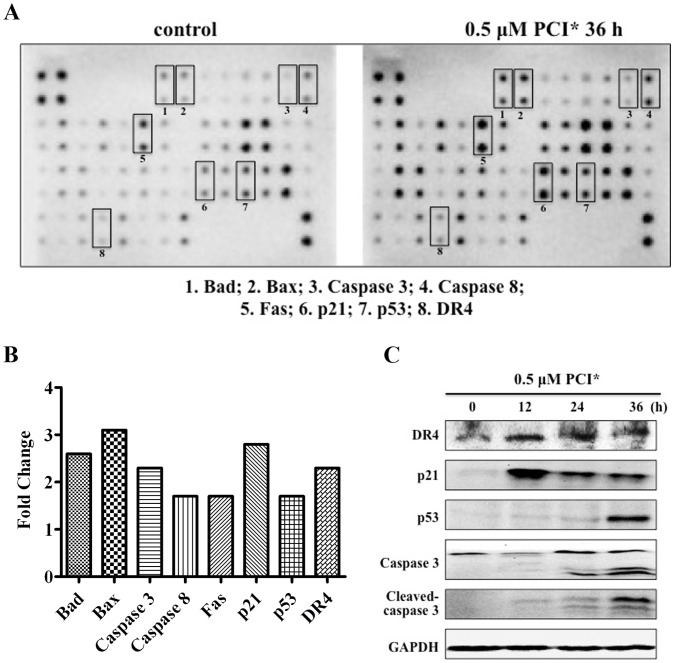
PCI-24781 affected both extrinsic and intrinsic apoptotic pathways. (A) SK-N-DZ cells were untreated (control) or treated with 0.5 µM of PCI-24781 for 36 h and the cell lysates were incubated with human apoptosis arrays according to the manufacturer’s instructions. Each protein was spotted in duplicate. The spots in upper left and lower right corner are positive controls. Eight apoptotic proteins were shown below. (B) Densitometric analysis was done by ImageJ software and the fold changes of apoptotic proteins were represented by histogram. (C) The expression of DR4, p21, p53, caspase 3 and cleaved caspase 3 was confirmed by Western blotting. GAPDH was used as loading control.

Previous studies have shown that HDAC inhibitors induced caspases dependent apoptosis by activation of the intrinsic apoptotic pathway in various tumors, including neuroblastoma cells [31]. We then investigated the expression of apoptosis-related proteins induced by PCI-24781 in SK-N-DZ cells. After 0.5 µM of PCI-24781 exposure for 36 h, cell lysates were analyzed by RayBio Human Apoptosis Antibody Array kit. A comparison of non-treated control and treated cells indicated that expression of most apoptotic proteins were increased, including Bad, Bax, caspase 3, caspase 8, Fas, p21, p53 and DR4 (Figure 3A). Quantification of protein signals by ImageJ software revealed that Bax increased 3.1-fold, Bad increased 2.6-fold, both caspase 3 and DR4 increased 2.3-fold, p21 increased 2.8-fold, and both caspase 8, Fas and p53 increased 1.7-fold (Figure 3B). To confirm these results, we detected the expression of p21, p53, caspase 3, cleaved-caspase 3 and DR4 by Western blotting analysis in SK-N-DZ cells. As shown in Figure 3C, PCI-24781 activated the expression of DR4, one death-inducing receptor which is known to play a key role in extrinsic apoptotic pathway. For cyclin-dependent kinase inhibitor p21, the most significant increase was observed after 12 hours of PCI-24781 treatment in SK-N-DZ cells. However, the expression of p53 was only up-regulated after 36 hours of PCI-24781 treatment. Additionally, PCI-24781 also induced cleavage of caspase 3, and the 19-kDa and 17-kDa cleaved products were present after PCI-24781 treatment for 24 h and 36 h. Taken together, these results suggest that the agent PCI-24781 could cause cell cycle arrest at G2/M phase and activate both extrinsic and intrinsic apoptotic pathway in SK-N-DZ cells.

### Proteomic Analysis Revealed Different Protein Expression Profiles in PCI-24781 Treated SK-N-DZ Cells

**Figure 4:**
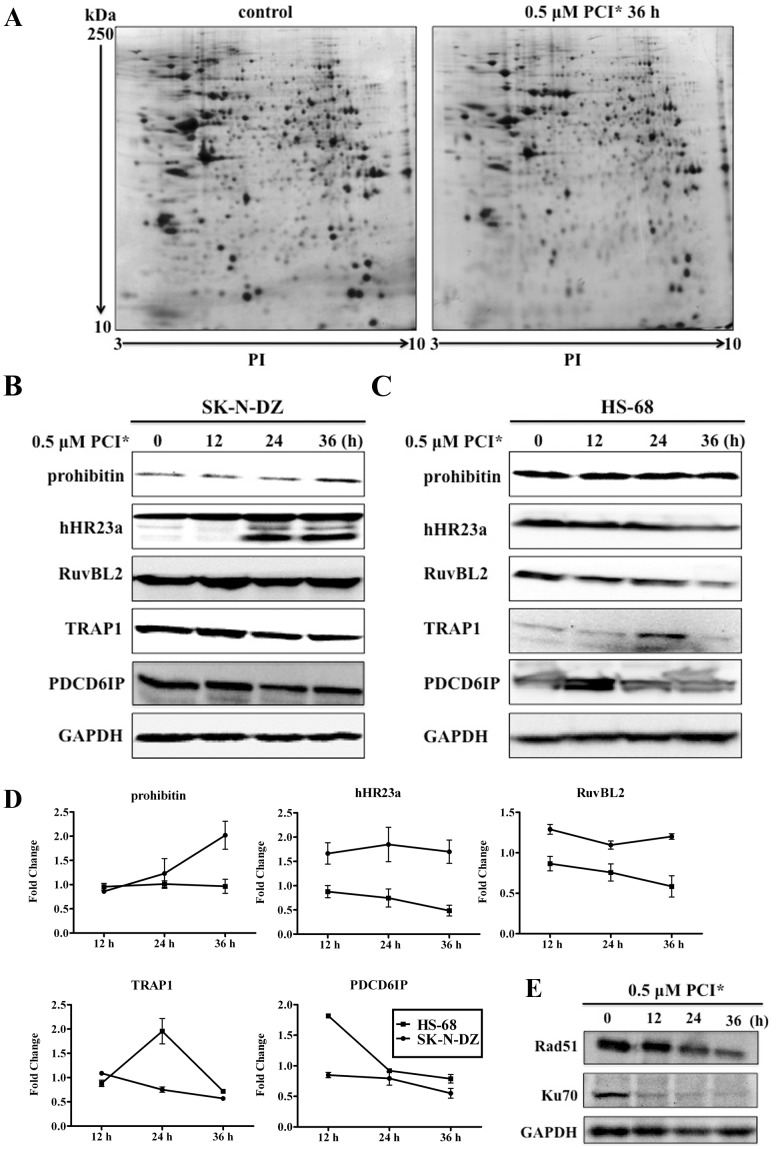
Proteomic analysis of protein expression patterns between control and PCI-24781-treated SK-N-DZ cells. (A) Representative 2-DE images of the control and cells treated for 36 h with 0.5 µM PCI-24781. Total proteins (500 µg) extracted from non-treated and treated cells were subjected to pH 3–10 nonlinear IPG strips and then separated by 12% SDS-PAGE. Total protein spots were visualized by CBB staining. The approximate isoelectric points (PI) and molecular mass (kDa) is shown at bottom and left of images, respectively. (B) Confirmation of expression of prohibitin, hHR23a, RuvBL2, TRAP1 and PDCD6IP in SK-N-DZ cells. Cells were treated with or without 0.5 µM PCI-24781 and then collected for Western blotting analysis as the indicated time points. (C) The effect of PCI-24781 on expression of the five selected proteins was valuated in HS-68 cells. The same treatment was performed as in SK-N-DZ cells. GAPDH was used as loading control. PCI*: PCI-24781. (D) Densitometry from Western blotting for SK-N-DZ cells and HS-68 cells following 12, 24 and 36 h treatment. Results reflect the mean ± SD for three independent experiments. (E) Total protein (40 µg) from SK-N-DZ cells untreated or treated with PCI-24781 was detected with specific antibodies for Rad51 and Ku70. GAPDH was used as loading control. PCI*: PCI-24781.

To examine the effect of PCI-24781 on protein expression in SK-N-DZ cell line, the differentially expressed protein profiles were analyzed by 2D electrophoresis. Representative 2-DE maps are shown in Figure 4A. After analysis of the 2D gel replicates by ImageMaster™ software, a total of 42 protein spots with differential expression were identified by MALDI-TOF MS system. The identified protein spots include 26 down-regulated and 16 up-regulated proteins, and the detailed information of these proteins, including PI, MW, Mascot score, C.I.%, etc., are listed in Table 1. They were further classified into functional groups according to the UniProtKB/Swiss-Prot database. The cluster analysis revealed that PCI-24781 affected a series of cellular processes, including cytoskeleton organization, energy metabolism, DNA repair, cell cycle, transcriptional regulation, etc. There are six proteins involving in cell cycle, proliferation and apoptosis processes. Of these, two proteins (prohibitin and calreticulin precursor) were up-regulated and others (PDCD6IP, HSP75, GGCT and TER ATPase) were down-regulated after PCI-24781 treatment. One DNA repair protein called hHR23a was found to increase in response to PCI-24781. Three proteins were associated with signal transduction and transcriptional regulation. From which, Rab GDI beta and HSP cognate 71 kD protein was down-regulated. While an ATPase protein RuvBL2, which has been shown to involve in many cellular pathways, was up-regulated after treatment. Heat shock proteins (HSPs) are a family of conserved chaperones that involved in maintaining cellular protein homeostasis. There are totally five heat shock proteins identified in this study, including HSP70 protein 4, HSP90 beta, HSP75, GRP78 and HSP cognate 71. Except for HSP70 protein 4, other four HSPs were down-regulated in response to PCI-24781. Most proteins that regulate metabolism were decreased by PCI-24781. Additionally, many identified proteins were located in mitochondria, such as prohibitin, HSP75 (TRAP1), citrate synthase, ATP5A1 and ATP-specific succinyl-CoA synthetase, indicating dysfunctional mitochondrion triggered by PCI-24781.

**Table 1:** The differentially expressed proteins regulated by PCI-24781 in SK-N-DZ cells (Average ratio ≥1.5 and ≤0.5).

Function	SpotNo.	Protein Name	AccessionNo.	PI/MW (kD)	No. ofPeptides	MascotScore	C.I. %	Fold
**Cytoskeleton organization**								
	1	Spectrin alpha	gi|154759259	5.22/284.4	37	106	100	1.67
	2	Tropomodulin	gi|6934244	5.08/39.6	12	80	100	0.24
	3	Fascin	gi|13623415	6.84/54.5	17	119	100	0.48
	4	Tropomyosin	gi|42476296	4.66/32.8	9	178	100	0.50
**Energy metabolism**								
	5	ATP-specific succinyl-CoA synthetase	gi|3766197	5.84/46.4	15	144	100	1.68
	6	Glucosidase 2 subunit beta isoform 2	gi|48255891	4.34/59.1	10	99	100	1.46
	7	Dihydrolipoamidedehydrogenase	gi|1339989	7.21/54.3	12	155	100	0.36
	8	Citrate synthase	gi|48257138	6.49/45.6	10	190	100	0.54
	9	Methylenetetrahydrofolate dehydrogenase	gi|14602585	6.75/101.5	36	273	100	0.18
	10	TXNDC4	gi|37183214	5.13/46.9	13	89	100	0.52
**Protein folding, modification &degradation**								
	11	Heat shock 70 kDa protein 4	gi|38327039	5.11/94.3	19	144	100	2.09
	12	GRP78	gi|386758	5.03/72.1	22	270	100	0.06
	13	Ubiquitin-activating enzyme E1	gi|23510338	5.49/117.8	22	249	100	0.48
	14	Heat shock protein 90-beta	gi|20149594	4.97/83.2	20	86	100	0.41
	15	Stress induced phosphor-protein1	gi|5803181	6.4/62.6	30	190	100	0.42
**DNA repair**								
	16	hHR23a	gi|38492677	4.56/40.0	9	106	100	1.57
**Cell cycle, proliferation & apoptosis**								
	17	Prohibitin	gi|46360168	5.57/29.8	15	525	100	2.12
	18	PDCD6IP	gi|22027538	6.13/96.0	29	303	100	0.50
	19	Calreticulin precursor	gi|4757900	4.29/48.1	13	141	100	0.87
**Function**	**Spot** **No.**	**Protein Name**	**Accession No.**	**PI/MW (kD)**	**No. of** **Peptides**	**Mascot Score**	**C.I. %**	**Fold**
								
	20	GGCT	gi|13129018	5.07/21.0	10	86	100	0.48
	21	TER ATPase	gi|6005942	5.14/89.3	30	194	100	0.48
	22	Heat shock protein 75 kD	gi|155722983	8.3/80.1	33	482	100	0.42
**RNA splicing & processing**								
	23	hnRNP M	gi|14141152	8.84/77.5	22	141	100	2.78
	24	KHSRP	gi|17402900	7.18/67.5	24	371	100	0.32
	25	hnRNP H3 isoform b	gi|14141159	6.36/35.2	15	245	100	0.38
	26	DDX1	gi|4826686	6.81/82.4	25	166	100	0.23
	27	KHSRP	gi|54648253	8/72.9	23	166	100	0.36
**Signal transduction & transcription regulation**								
	28	RuvB-like 2	gi|5730023	5.49/51.1	25	367	100	1.47
	29	Rab GDI beta	gi|169646441	5.91/45.6	20	233	100	0.55
	30	Heat shock cognate 71 kDa	gi|5729877	5.37/70.9	26	363	100	0.05
**Amino acid & nucleic acid metabolism**								
	31	xaa-Pro dipeptidase	gi|149589008	5.64/54.5	12	181	100	1.78
	32	PSAT1	gi|16741698	7.56/40.4	16	123	100	0.40
	33	KIAA0361	gi|2224663	5.57/148.0	25	185	100	0.30
	34	PURH	gi|20127454	6.27/64.6	26	223	100	0.42
**Protein biosyhthesis**								
	35	Nucleolin	gi|189306	4.59/76.3	21	352	100	1.98
	36	Elongation factor2	gi|4503483	6.41/95.3	24	195	100	0.05
	37	Tyrosyl-tRNA synthetase	gi|4507947	6.61/59.1	26	273	100	0.50
**Electron transport**								
	38	ATP5A1	gi|15030240	9.07/59.8	21	429	100	2.93
	39	Cytochrome b-c1 complex subunit 5	gi|163644321	8.55/29.6	10	223	100	1.89
**Others**								
Blood pressure	40	Alloalbumin	gi|178345	5.99/69.2	7	120	100	1.65
Blood pressure	41	Albumin-like	gi|763431	5.69/52.0	8	99	100	1.50
Structural of ribosome	42	28S ribosomal protein S22	gi|9910244	7.7/41.3	19	337	100	2.53

### Western Blotting Validated the Identified Proteins in SK-N-DZ Cells

To validate the findings obtained from 2-DE experiment, the expression level of five candidate proteins (prohibitin, hHR23a, RuvBL2, TRAP1, PDCD6IP) were further investigated by immunoblotting analysis. As shown in Figure 4B and D, the corresponding changes in protein expression are basically consistent with the results obtained from 2-DE, with the exception of RuvBL2 that was slightly increased upon PCI-24781 treatment. High expression level of prohibitin was only observed at 36 h after PCI-24781 treatment in SK-N-DZ cells. PCI-24781 induced increased expression in a time-dependent manner for hHR23a, known for characterized function in nucleotide excision repair (NER). Interestingly, a fragment of about 35 kDa was only markedly induced after PCI-24781 treatment for 24 and 36 h, which might be the isoform of hHR23a according to its molecular weight. It’s been reported that hHR23a gene has different isoforms based on alternative splicing at transcriptional level [32]. RuvBL2 is an ATP-binding protein that belongs to AAA+ (ATPases associated with diverse cellular activities) ATPase. RuvBL2 was identified in various chromatin-remodeling complexes, such as TIP60/NuA4 (nucleosomal acetyltransferase of H4) complex, thus involved in many cellular pathways [33]. In SK-N-DZ cells, the expression of RuvBL2 was just slightly increased after PCI-24781 treatment. However, PCI-24781 inhibited the expression of TRAP1 and PDCD6IP, which were related with anti-apoptosis and proliferation, respectively (Figure 4B). HSP75, also known as TRAP1 (tumor necrosis factor receptor-associated protein 1), is involved in the protection from DNA damage and apoptosis in mitochondrion [34]. While studies have shown that overexpression of PDCD6IP (programmed cell death 6-interacting protein) could block apoptosis [35]. Therefore, the down-regulation of TRAP1 and PDCD6IP in SK-N-DZ cells might partially contribute to cell death caused by PCI-24781. In addition, there are other two DNA repair proteins Rad51 and Ku70 that play roles in cell death induced by HDAC inhibitors [25], [26], [36]. For example, Rad51 associated with homologous recombination (HR) was inhibited by PCI-24781 in HCT116 cells [36]. Ku70, as mentioned before, involved in Bax-induced cell death in neuroblastoma cells [25], [26]. Based on these findings, we also analyzed the expression level of the two proteins (Rad51 and Ku70) after PCI-24781 treatment in SK-N-DZ cells. Consistent with previous reports, PCI-24781 indeed decreased the expression level of Rad51 after treatment for 24 and 36 h, while Ku70 was significantly inhibited at the beginning of 12 h of treatment (Figure 4E).

### The Five Selected Proteins were Differentially Regulated in HS-68 Cells After PCI-24781 Treatment

The relative resistance to PCI-24781 in HS-68 cells triggered us to dissect more detailed mechanism of PCI-24781. The expression pattern of the above five candidate proteins was totally different in HS-68 cells, when compared to SK-N-DZ cells (Figure 4C and D). There were no changes for prohibitin expression after the PCI-24781 treatment. PCI-24781 decreased the expression of both hHR23a and RuvBL2 in HS-68 cells. Although PDCD6IP and TRAP1 proteins had an increased expression level after treatment for 12 h and 24 h, respectively, the HS-68 cells subsequently restored their expression to normal levels.

Altogether, these results suggest a more detailed mechanism of PCI-24781 in neuroblastoma cells. PCI-24781 exposure led to up-regulation of some anti-proliferation proteins such as prohibitin, and down-regulation of some anti-apoptosis proteins such as TRAP1 and PDCD6IP in sensitive SK-N-DZ cells. However, no changes in the expression of prohibitin were detected, as well as reduction of TRAP1 and PDCD6IP in non-sensitive HS-68 cells. In addition, high abundance of hHR23a and RuvBL2 was found in SK-N-DZ cells, but the inhibition of their expression was detected in HS-68 cells after treatment. We thus assumed that hHR23a and/or RuvBL2 may account for PCI-24781-induced cell death in SK-N-DZ.

### Deletion of RuvBL2 Attenuated Cell Death Caused by PCI-24781 in SK-N-DZ Cells

**Figure 5:**
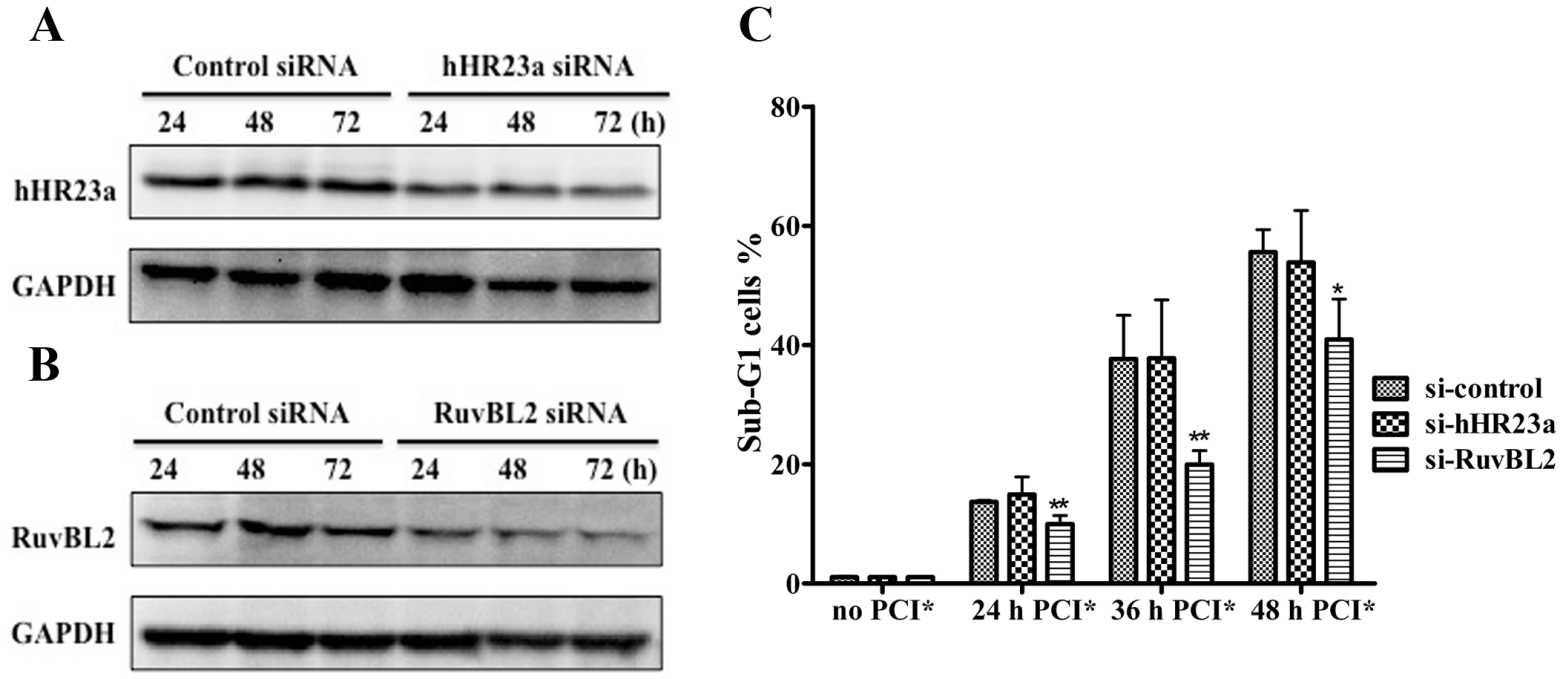
Functional involvement of hHR23a and RuvBL2 in PCI-24781 mediated cell death. SK-N-DZ cells were transfected with 5 nM control siRNA or specific siRNA against hHR23a and RuvBL2 for 24 h, 48 h and 72 h. Protein levels of hHR23a (A) and RuvBL2 (B) were analyzed by Western blotting. GAPDH was used as loading control. (C) Cells transfected with 5 nM hHR23a, RuvBL2 and control siRNA for 48 h were then exposed to no or 0.5 µM PCI-24781 for the indicated times. The percentage of sub-G1 cells was analyzed by flow cytometry. Error bars represent the means ± S.D. of three independent experiments. *P*<0.05 and *p*<0.01 are indicated by * and **, respectively.

To ascertain if this increase in hHR23a and RuvBL2 was functionally important for PCI-2481-mediated cell death, we used siRNA method to inhibit the expression of hHR23a and RuvBL2 gene and then valuated the effect on apoptosis after PCI-24781 treatment in SK-N-DZ cells. As shown in Figure 5A and B, when compared to control siRNA, both hHR23a and RuvBL2 protein expression was significantly decreased in a time-dependent manner after specific siRNA transfection. Cells transfected with siRNA for 48 h were then exposed to PCI-24781 for 24 h, 36 h and 48 h. Analysis of apoptotic sub-G1 cells showed that RuvBL2 partially protected cells from PCI-24781-induced cell death, while hHR23a showed similar changes with control siRNA (Figure 5C). Importantly, we found that cells transfected with RuvBL2 conferred the maximum protection after PCI-24781 treatment for 36 h. The percentages of sub-G1 cells were 37.7%, 37.8% and 19.9% in cells transfected with control, hHR23a and RuvBL2, respectively, after PCI-24781 treatment for 36 h (Table S1). These results demonstrate that the depletion of RuvBL2, not hHR23a attenuates the sensitivity of SK-N-DZ cells to PCI-24781-induced cell death, indicating that RuvBL2 is a key target protein responsible for PCI-24781 antitumor activity in SK-N-DZ cells.

## Discussion

HDAC inhibitors are considered as one of the most promising epigenetic treatments for cancer, and multiple preclinical and clinical data achieved significant success in a wide range of tumor types due to the acceptable side effects [6]. Therefore, understanding the tumor response to HDAC inhibitors and their roles in anti-tumor activity may help to develop more effective clinical protocols. Herein, we have evaluated the effect on growth and survival by two HDAC inhibitors PCI-24781 and CI-994 in three human neuroblastoma cell lines SK-N-DZ, SH-SY-5Y and SK-N-SH, and further investigated the detailed mechanisms of PCI-24781 in SK-N-DZ cell line. PCI-24781 belongs to hydroxamic acid-based HDAC inhibitor and is able to inhibit both class I (HDAC 1, 2, 3, 8) and class II (HDAC 4, 5, 7, 9, 6, 10) HDACs [28]. However, CI-994 is a benzamide derivative that has been shown to inhibit class I HDACs [30]. We showed that these cell lines exhibited different sensitivity to PCI-24781 and CI-994. SK-N-DZ was the most sensitive cell line and SH-SY-5Y was less sensitive in response to PCI-24781 for 24 h. HS-68 and SK-N-SH shared the similar sensitivity to PCI-24781. However, treatment with PCI-24781 for 48 h can significantly decrease the cell viability in SK-N-SH but not in HS-68 (Figure S1). Although CI-994 treatment at 24 h also decreased the cell viability in a dose-dependent manner in all the cell lines, this effect was evident in HS-68 but slight in three other NB cell lines among the concentrations of 0.03–3 µM CI-994. The higher doses of CI-994 (3–30 µM) significantly decreased the cell viability only in SK-N-DZ but not in HS-68, as well as SH-SY-5Y and SK-N-SH. The different sensitivity to a single HDAC inhibitor may be caused by different genetic background among neuroblastoma cell lines. In contrast to SK-N-SH and SH-SY-5Y cells with N-myc single copy, SK-N-DZ is a N-myc amplified neuroblastoma cell line. Moreover, SK-N-SH and SH-SY-5Y cells are hyperdiploid cell line with chromosome number of 47, while SK-N-DZ cells have the chromosome number of 44 due to the loss of both copies of chromosome 2 [4], [37]. Indeed, previous studies have shown that the N-myc amplified neuroblastoma cell lines (SMS-KCNR, LA1-55N and SK-N-JD) were more sensitive to HDAC inhibitors (MS-27-275 and TSA) than the unamplified neuroblastoma cell line (SK-N-AS) [21], [38]. In addition, PCI-24781 exhibited stronger anti-tumor activity at very low doses when comparing with CI-994. The reason may be the fact that PCI-24781 has a broader spectrum of activity and could inhibit class I and class II HDACs. While CI-994 could only interfere class I HDACs. These results provide a clue that the choice of clinical protocols, including the duration and doses of administration, varies depending on different background of patients.

We then showed that PCI-24781 induced cell cycle arrest in G2/M phase and accumulation of sub-G1 cells in a time- and dose- dependent manner in SK-N-DZ cell line. However, no evident effect can be observed in HS-68 cell line, strongly indicating that PCI-24781 has less cytotoxicity in the normal cell line HS-68. The cell cycle arrest induced by HDAC inhibitors has been described in many types of tumors, including neuroblastoma cell lines [19], [20], [21], [39]. For example, HDAC inhibitors such as TSA, SAHA and NaB were shown to induce G2/M cell cycle arrest in neuroblastoma cells [19]. In addition, PCI-24781 was reported to mediate the cell cycle arrest in G0/G1 phase in lymphoma cells [40] while in G2/M phase in soft tissue sarcoma [41]. Altogether these findings indicate that the effects on cell cycle progression by HDAC inhibitors depend on tumor types and compounds studied.

We also demonstrated that PCI-24781 activated both extrinsic and intrinsic apoptotic pathway in SK-N-DZ cells. After treatment with PCI-24781, the death receptor (DR4) was increased and then interacted with downstream effectors of signal transduction, finally leading to the activation of caspase 3 dependent apoptosis. Our results also support this conclusion, as activation of DR4 is an earlier event than that of caspase 3. This is in accordance with previous studies reporting that both extrinsic and intrinsic apoptotic pathway involved in cell death induced with HDAC inhibitors, but the activation of certain signal pathways in HDAC inhibitors-induced apoptosis was shown to vary depending on tumor types and compounds studied [5], [15], [24]. In addition to apoptosis, the induction of autophagy by HDAC inhibitors was recently proposed in neuroblastoma cells [21]. In this study, the cyclin-dependent kinase inhibitor p21 and the tumor suppressor gene p53 were also increased by PCI-24781. However, in contrast to p21 with high expression level between 12 h and 36 h of treatment, the increased expression of p53 occurred only after 36 h treatment with PCI-24781. This suggests that p21 may play its role in a p53-independent manner. As the most common target gene of HDAC inhibitors, p21 was associated with cell cycle arrest both in G1 and G2 phase [42]. Thus p21 may contribute to G2/M phase arrest observed in SK-N-DZ cells treated by PCI-24781.

Interestingly, although no cell cycle arrest and evident sub-G1 peak can be observed in HS-68 cell line, treatment with PCI-24781 induced accumulation of acetylated histone H3 in HS-68 cells, as well as SK-N-DZ cells. This suggests that hyper-acetylation caused by HDAC inhibitors is not indispensable part for HDAC inhibitor-induced cell death, implying that normal cells, not like cancer cells, may have their own resistant system to protect cells from apoptosis in response to HDAC inhibitors. Previous studies have shown that HDAC inhibitors were selectively cytotoxic to cancer cells but relatively non-toxic to normal cells [5], [6]. One of the reasons may be that the G2 checkpoint was defective in cancer cells but not in normal cells after HDAC inhibitor treatment [43]. However, more detailed information is still unclear. Thus, to further explore the mechanism of action of PCI-24781, we analyzed the differentially expressed proteins between non-treated and treated cells using 2DE-based proteomic method. Proteomic analysis in this study revealed a total of 42 differentially expressed proteins, which involved in diverse biological processes such as metabolism, protein folding, signal transduction, DNA repair, transcriptional regulation and proliferation. As previously described disruption of mitochondria and metabolism [44], we also identified many proteins associated with mitochondrial function, including prohibitin, HSP75 (TRAP1), citrate synthase, ATP5A1 and ATP-specific succinyl-CoA synthetase. Prohibitin is a pleiotropic protein that plays a key role in proliferation, transcription and mitochondrial protein folding [45]. It has been reported that prohibitin can prevent cell proliferation and mediate transcriptional repression that required HDAC activity [46]. Therefore, the up-regulation of prohibitin in SK-N-DZ cells after PCI-24781 treatment may partially explain the reduction of proliferation. HSP75, also named as TRAP1, is a mitochondrial antiapoptotic heat-shock protein [34]. Its down-regulation can also partially explain the induction of apoptosis in SK-N-DZ cells after treatment with PCI-24781. We also identified other four heat shock proteins in our study, including HSP70 protein 4, HSP90 beta, GRP78 and HSP cognate 71. It has been reported that HDAC can also target many non-histone proteins such as HSPs. The hyperacetylation of HSP90 caused by HDAC inhibitors has been demonstrated to inhibit its chaperone function [47]. Moreover, depletion of GRP78 levels or inhibition of their functions by hyperacetylation was shown to induce autophagy in response to panobinostat treatment [48]. Similar to previous studies reporting that HSP27 was found to be down-regulated by HDAC inhibitors such as SAHA and FK228 [49], [50], PCI-24781 also reduced the expression of these HSPs identified from this study, except for HSP70 protein 4. Thus the down-regulation of these HSPs proteins may facilitate the cell death induced by PCI-24781 via disruption of protein homeostasis. However, further work is needed to confirm their roles in PCI-24781-mediated cell death.

Previous studies have shown that HDAC inhibitors may act, in part, via inhibiting genes associated with DNA repair such as Rad51 and Ku70 [25], [36]. However, we found that PCI-24781 induced high expression level of hHR23a that plays a role in NER. We thus measured the expression level of Rad51 and Ku70 in SK-N-DZ cells after PCI-24781 treatment, and found that PCI-24781 indeed triggered a significant reduction of Rad51 and Ku70. This differential expression pattern among DNA repair proteins may be explained by the fact that hHR23a also involved in ubiquitin-mediated protein degradation, in addition to its function in NER [51]. In our study, we also identified a protein called PDCD6IP, and its overexpression has been shown to block apoptosis [35]. Thus down-regulation of PDCD6IP may play a role in PCI-24781-mediated growth inhibition of SK-N-DZ cells, as well as induction of apoptosis. Another interesting finding in our current study was that PCI-24781 induced high abundance of RuvBL2 in SK-N-DZ cells. RuvBL2 is an AAA+ ATPase associated with the assembly of several complexes such as TIP60 histone acetyltransferase complex, and thus involves in chromatin remodeling, transcriptional regulation, DNA repair and telomerase activity [33], [52], [53]. It had been reported that RuvBL2 and its homology could cooperate with a repressor complex c-myc/Miz-1 to regulate the expression of p21 [33]. In addition, RuvBL2 was shown to affect Rad51 recruitment to chromatin in response to DNA damage [54].

We then confirmed the expression level of five candidate proteins (prohibitin, hHR23a, RuvBL2, TRAP1, PDCD6IP) in SK-N-DZ cells by Western blotting analysis. Additionally, these candidate proteins were also detected in HS-68 cells. Interestingly, PCI-24781 had no effect on expression of prohibitin but decreased the expression of both hHR23a and RuvBL2 in HS-68 cells. Moreover, PCI-24781 induced an increased expression of TRAP1 and PDCD6IP during treatment from 12 h to 36 h. Thus, it is reasonable to infer that down-regulation of hHR23a and RuvBL2 and up-regulation of TRAP1 and PDCD6IP may facilitate HS-68 cells to escape apoptosis from PCI-24781 treatment. To further investigate the roles of hHR23a and RuvBL2 in PCI-24781-mediated cell death in SK-N-DZ cells, we interfered their expression by siRNA and detected the sensitivity of SK-N-DZ cells to PCI-24781. Our results demonstrated that RuvBL2, not hHR23a could partially protect SK-N-DZ cells from PCI-24781-induced cell death.

**Figure 6:**
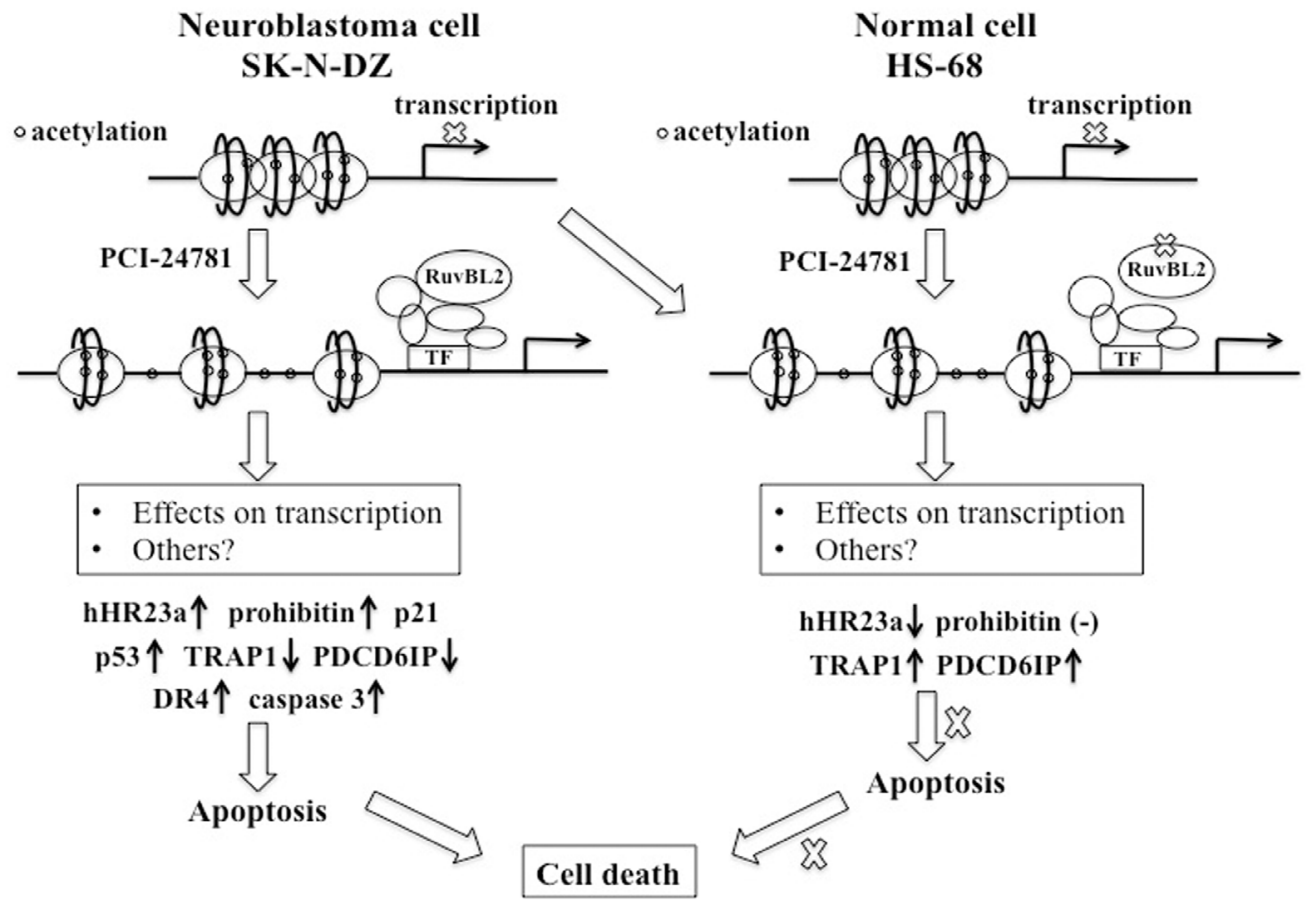
Schematic representation of proposed mechanism involved in PCI-24781-induced cell death. Proteins with up arrow and down arrow represent up-regulation and down-regulation, respectively in response to PCI-24781 treatment. Symbol (−) represents no change upon PCI-24781 treatment. TF: transcription factor in the chromatin complex.

In conclusion, we propose a novel mechanism of the action of PCI-24781 (Figure 6). Briefly, these findings indicate that PCI-24781 induces hyperacetylation in neuroblastoma cells SK-N-DZ and normal cells HS-68, but normal cells have the capacity to differentially regulate gene expression that finally block cell death. Moreover, PCI-24781-induced cell death in SK-N-DZ cells is in part due to the high abundance of RuvBL2 not hHR23a. This may be explained by the fact that redundant roles of other proteins associated with DNA repair and protein degradation such as HSPs can be found in cells. Our results therefore suggest that RuvBL2 is a key target protein upon PCI-24781 treatment in SK-N-DZ cells. Based on previous reports about RuvBL2, we speculate that knockdown of RuvBL2 may indirectly affect downstream gene expression via interfering the assembly of chromatin-remodeling complexes such as TIP60 histone acetyltransferase complex, which finally blocking PCI-24781-induced cell death. In summary, our current studies highlight the role of RuvBL2 in PCI-24781-induced cell death in SK-N-DZ cells. However, whether RuvBL2 plays a key role in antitumor activity of other HDAC inhibitors still need to be further evaluated. Additionally, the mechanism behind differential gene activation upon HDAC inhibitors in HS-68 cells is also unclear. Only better understanding of action of HDAC inhibitors can guide us to use these drugs in a more efficient manner for cancer therapy.

## Supporting Information

Figure S1 **Cell viability of PCI-24781 treatment for 48 h.** Treatment with PCI-24781 for 48 h significantly inhibited the growth of neuroblatoma cell line SK-N-SH, but normal cell line HS-68 showed relative resistance upon PCI-24781 treatment for 48 h.
(TIF)

Table S1 **Percentage of sub-G1 cells for siRNA assay.** Cells were transfected with 5 nM hHR23a, RuvBL2 and control siRNA for 48 h, respectively, and then treated with or without 0.5 µM PCI-24781 for 24 h, 36 h and 48 h. The values represent the average of three biological replications. PCI*: PCI-24781.
(DOC)
