# Supplementary figures and images for: RuvBL2 Is Involved in Histone Deacetylase Inhibitor PCI-24781-Induced Cell Death in SK-N-DZ Neuroblastoma Cells
Source: PLoS One. 2013 Aug 16;8(8):e71663. doi: 10.1371/journal.pone.0071663 (PMC3745445; doi:10.1371/journal.pone.0071663)

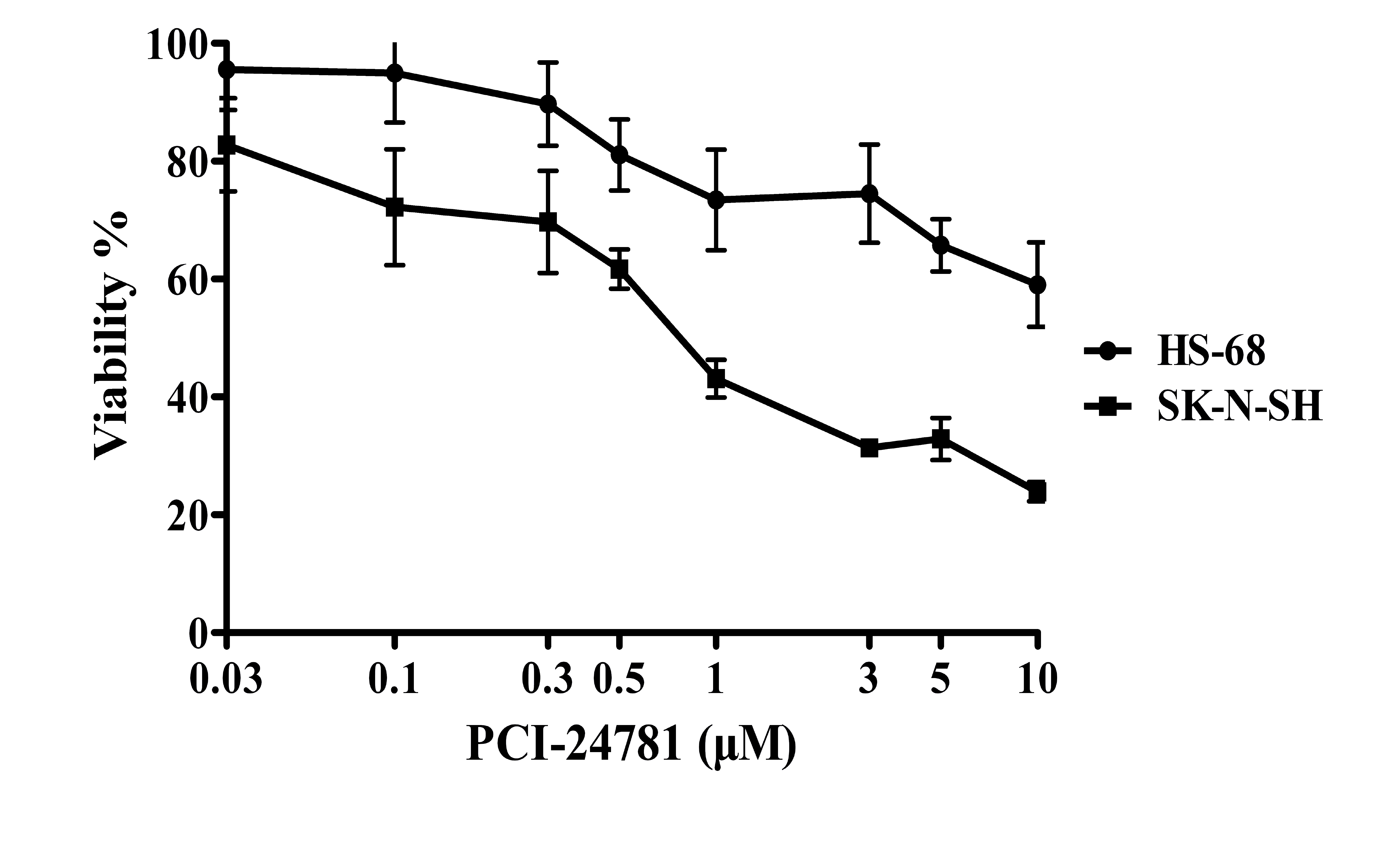

Supplement: Figure S1 — Cell viability of PCI-24781 treatment for 48 h. Treatment with PCI-24781 for 48 h significantly inhibited the growth of neuroblatoma cell line SK-N-SH, but normal cell line HS-68 showed relative resistance upon PCI-24781 treatment for 48 h. (TIF) [file pone.0071663.s001.tif]
