# Supplementary material for: RuvBL2 Is Involved in Histone Deacetylase Inhibitor PCI-24781-Induced Cell Death in SK-N-DZ Neuroblastoma Cells
Source: PLoS One. 2013 Aug 16;8(8):e71663. doi: 10.1371/journal.pone.0071663 (PMC3745445; doi:10.1371/journal.pone.0071663)
Supplement: Table S1 — Percentage of sub-G1 cells for siRNA assay. Cells were transfected with 5 nM hHR23a, RuvBL2 and control siRNA for 48 h, respectively, and then treated with or without 0.5 µM PCI-24781 for 24 h, 36 h and 48 h. The values represent the average of three biological replications. PCI*: PCI-24781. (DOC) [file pone.0071663.s002.doc]

**Table S1**. Percentage of sub-G1 cells for siRNA assay. Cells were transfected with 5 nM hHR23a, RuvBL2 and control siRNA for 48 h, respectively, and then treated with or without 0.5 µM PCI-24781 for 24 h, 36 h and 48 h. The values represent the average of three biological replications. PCI*: PCI-24781.

| sub-G1 cells% | no PCI* | 24 h PCI* | 36 h PCI* | 48 h PCI* |
| --- | --- | --- | --- | --- |
| si-control | 1.1 | 13.7 | 37.7 | 55.6 |
| si-hHR23a | 1.1 | 14.9 | 37.8 | 53.9 |
| si-RuvBL2 | 1.1 | 9.9 | 19.9 | 40.9 |
